# HIV Reservoirs and Immune Surveillance Evasion Cause the Failure of Structured Treatment Interruptions: A Computational Study

**DOI:** 10.1371/journal.pone.0036108

**Published:** 2012-04-27

**Authors:** Emiliano Mancini, Filippo Castiglione, Massimo Bernaschi, Andrea de Luca, Peter M. A. Sloot

**Affiliations:** 1 University of Amsterdam, Computational Science, Amsterdam, The Netherlands; 2 Institute for Computing Applications Mauro Picone, National Research Council, Roma, Italy; 3 Institute of Clinical Infectious Diseases, Catholic University, Roma, Italy; 4 Infectious Diseases Unit, Siena University Hospital, Siena, Italy; 5 National Research University ITMO, Information Technologies, Mechanics and Optics, St. Petersburg, Russia; 6 Nanyang Technological University, Singapore, Singapore; Dana-Farber Cancer Institute, United States of America

## Abstract

Continuous antiretroviral therapy is currently the most effective way to treat HIV infection. Unstructured interruptions are quite common due to side effects and toxicity, among others, and cannot be prevented. Several attempts to structure these interruptions failed due to an increased morbidity compared to continuous treatment. The cause of this failure is poorly understood and often attributed to drug resistance. Here we show that structured treatment interruptions would fail regardless of the emergence of drug resistance. Our computational model of the HIV infection dynamics in lymphoid tissue inside lymph nodes, demonstrates that HIV reservoirs and evasion from immune surveillance themselves are sufficient to cause the failure of structured interruptions. We validate our model with data from a clinical trial and show that it is possible to optimize the schedule of interruptions to perform as well as the continuous treatment in the absence of drug resistance. Our methodology enables studying the problem of treatment optimization without having impact on human beings. We anticipate that it is feasible to steer new clinical trials using computational models.

## Introduction

The increase in life expectancy of HIV positive individuals raised both costs and side effects of combination Anti-Retroviral Therapy (cART), stimulating research into Structured Treatment Interruptions (STI). Latest clinical trials on STI [1], [2] indicated they are less effective than continuous treatment. We show that the STI tested so far in clinical trials would fail regardless of the emergence of drug resistance and that this failure is caused by HIV reservoirs and immune evasion.

For many HIV positive individuals treatment interruptions are unavoidable. Although clinical studies proved that increased risks are associated to cART interruptions, patient-initiated unstructured treatment interruptions are still quite common in the clinical practice [3]. A recent systematic review [4] of cohort studies and clinical trials indicates a proportion of unstructured treatment interruptions ranging from 5.8% to 83.1% with a median of 23.1%. The mean duration of cART interruptions ranges from 11.5 days to 18 months with a median of 150 days. The main reported reasons for treatment interruptions are laboratory toxicity and clinical side effects. Less frequent arguments are patient compliance, treatment fatigue, intercurrent illness and other reasons.

The problem of unstructured interruptions is getting worse because of the increasing duration of treatment. Recent studies suggest an earlier use of cART as a way to fight effectively the HIV epidemics [5], [6]. Current cART guidelines defer the treatment to the time when CD4^+^ counts drop below 350 (European guidelines - EACS) or 500 cells per microliter (US guidelines - DHHS and IAS-USA) whereas recent studies indicate that an early start of cART (CD4^+^ counts >500 cells per microliter) could significantly improve survival [5], [7]–[9]. Regardless of the success of anti-retroviral therapies, HIV's ability to mutate and evade both antiviral treatments and vaccines shifted the attention from curing affected individuals to fighting the epidemics. Some predictive models investigate the effects of different strategies on the HIV epidemics [6], [10]. Recent clinical investigations [9] indicate that a more intensive and earlier use of cART is effective in reducing the spread of the virus. Indeed, another study highlighted the effectiveness of preventive use of cART in reducing the chance of being infected in case of sexual contact with a seropositive individual [11]. Strategies aimed at reducing the spread of infection not only extend the duration of cART for an infected individual but also increase the number of individuals simultaneously under cART, raising the - already high - global cost of cART treatments.

STI aimed at discontinuing the therapy according to a schedule so as to minimize the side effects without losing substantial protection. In a large, randomized clinical trial [1], [2] STI were associated with an increased risk of death and opportunistic diseases connected to treatment interruptions. Earlier studies [12], [13] indicated positive results for STI, in some cases associated to supporting drugs [14]. Subsequently, many clinical studies on different STI schedules resulted in generally neutral or negative outcomes [15]–[17], although the reasons are still not fully understood. Results from the *Staccato* randomized trial indicated substantial drug savings and did not result in increased drug resistance in the STI arm, while treatment related adverse events were more frequent with continuous treatment [18].

Hereafter we resort to a computational model to gain a better understanding of the reasons of STI failure. A number of mathematical models describe the HIV infection dynamics and the related immune response. Some of them take into account the use of cART [19]–[21]. Most mathematical models use a continuous description of time, allowing the use of optimal control techniques to search for the best time to deliver the therapy [22]–[24]. Recently a mathematical model searched for the underlying reasons of STI failure [25]. That model confirms that viral mutation and the emergence of drug resistant strains may be accelerated by STI, although only activated CD4^+^ cells and macrophages were considered as possible hosts for the virus. The study of HIV through cellular automata and agent-based models is also common [26]–[31] due to the discrete nature of the biological entities involved in the phenomena. In this work we use a well-established and validated agent-based model (ABM) of HIV infection [32]–[36]. See File S1 for a brief description of the model used in this work.

Our aim is to gain a better understanding of the reasons behind STI failure: are they inherent to the HIV dynamics or consequential of other mechanisms like the emergence of drug resistance? By excluding to model the resistance to the drugs we restrict the possible causes of STI failure to mechanisms such as virus reservoirs in macrophages and resting/memory CD4^+^ T lymphocytes. In addition, in this simplified formulation, we search for an optimal STI and compare it to the optimal one found in a previous work [32]. A similar attempt to optimize clinical treatments using *in silico* modeling has been applied to cancer-preventing vaccinations [37]. In that case the predictions of the computational model were validated through specific *in vivo* experiments on mice. That paper proved that an integrated *in vivo-in silico* approach is able to improve mathematical and biological models for cancer immunoprevention.

In the present work we investigate treatment interruptions with two *in silico* simulations: in the first simulation we test three STI used in clinical trials and compare their efficacy to that of the continuous (i.e., uninterrupted) treatment, while in the second simulation we search for an optimal STI scheduling and compare it with random treatment and continuous treatment. By ‘optimal schedule’ we mean the one that less impairs the ability to mount an immune response while keeping the amount of drugs used to a minimum. We use the Simulated Annealing (SA) technique to search for the optimal STI schedule. We simulate the disease progression for a group of 250 *virtual* (i.e., *in silico*) HIV positive patients. For each group of *virtual* patients we compare the effects of different treatment strategies on the HIV infection over a therapeutic period of 48 weeks, three years after seroconversion. We finally evaluate the efficacy of each STI schedule by challenging the immune system of the virtual HIV patients with a simulated opportunistic bacterial infection at the end of the treatment.

## Results

### Optimization results

**Figure 1:**
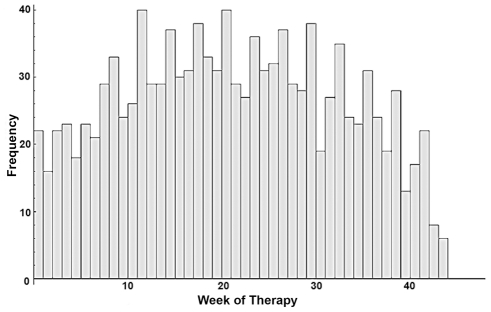
Optimized HAART Histogram: the histogram shows 50 optimized therapeutic schedules. Each bin represents the frequency of a given week of therapy in the optimized schedules.

**Figure 2:**
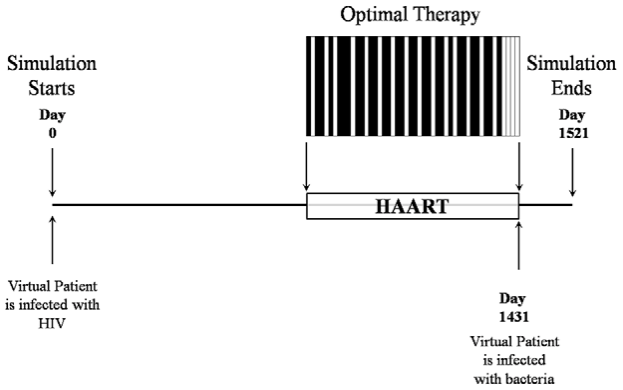
At time 0 a virtual patient is infected with HIV. The therapeutic period of 48 weeks starts after 3 years of untreated infection (day 1096). The optimal therapy is shown in the figure. At the end of the 48 weeks of therapy we inject a fixed amount of bacteria in the virtual patient, challenging his immune system. The patient dies if the antigens exceed a threshold of 4×10^6^ bacteria/ml. An efficient therapy is capable of restoring the patient's immune system enough to contain the bacterial infection.

To quantitatively evaluate the effect of a STI schedule of cART we define a fitness function that measures the health of the virtual patient's immune system and the amount of drugs received over the 48 weeks period. The fitness function is described in the Materials and Methods section. We performed 50 SA optimizations by using different initial conditions. These optimizations resulted in 50 optimized treatment schedules with an average fitness score of 2.325 and a standard deviation of 0.012. The optimal treatment schedules have 29 weeks of therapy on average and a standard deviation of 3 weeks. The optimized schedules are shown in a histogram (Figure 1) in which each bin represents the frequency of a given week of therapy in the optimized schedules. The histogram shows a peak every 3 weeks spaced out by 2 weeks in which the drug administration has a lower but still significant frequency. Due to the requirement of providing a schedule as regular as possible, we define as “optimal" a schedule having 2 weeks of therapy followed by an interruption of 1 week for 32 weeks after the initial two months. The optimization shows a different pattern for the initial two months of treatment (Figure 2). The optimal schedule has 29 weeks of therapy and is shown in Figure 2. According to our simulations the last 4 weeks of the therapeutic period have limited importance. In other words, the effect of cART at later times is somehow less important, supporting the current opinion that it is more rewarding to support the immune system with anti-retroviral treatment at earlier times.

**Figure 3:**
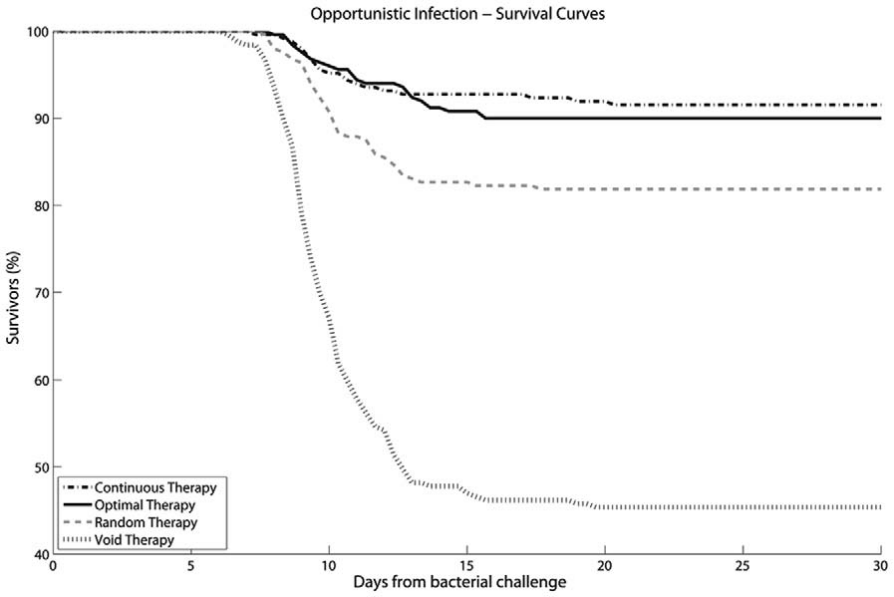
Survival curves: percentage of survivors over time in a population of 250 HIV+ virtual patients. 30 days after bacterial challenge survival rates are the following: continuous therapy 91.53%, optimal therapy 90%, random therapy 81.85% and void therapy 45.38%. A reduction of 40% of the drug intake corresponds to less than 5% reduction in the survival rate.

The aim of the treatment was to suppress the HIV viral load and induce immune restoration, resulting in an immune system capable of dealing with opportunistic infections. To assess the efficacy of the optimal therapy indicated by the SA algorithm, we ran simulations of an opportunistic infection starting immediately after the therapeutic period. Bacteria were injected in the virtual patients as soon as the 48 weeks of therapy were over. The immune system reaction against those bacteria depended on its efficiency at injection time. We compared the result of the schedule optimized by the SA algorithm with three different simulated control groups (Figure 3). In the first group (“Continuous Therapy") the drugs were administered every day for a therapeutic period of 48 weeks as prescribed by the cART guidelines; in the second group (“Random Therapy") the total amount of drug was equal to that of the optimal therapy but administration within the therapeutic period was performed randomly; finally, in the last group, there was no therapy (“Void Therapy"). To measure the effectiveness of the optimized STI we monitored the survival curve in a population of 250 virtual HIV+ patients infected by a bacterium 4 years after their initial HIV infection. For the same 250 patients we compared the effects of continuous therapy, void therapy, optimal therapy and random therapy.

Our results show that the optimal therapy provided a survival rate comparable to the continuous therapy using 40% less drug. Survival rate for untreated patients was 45.4%. The optimal therapy performed significantly better than the void one (survival rate 90% versus 45.4%). The optimal therapy also performed better than a random therapy with the same amount of drugs (a survival rate of 90% *vs.* 81.9%). The latter result indicates that the schedule identified by the SA algorithm is optimized for both the amount of medicine and the administration schedule, supporting the importance of finding a specific scheduling to reduce therapeutic failures.

### Comparison with clinical trials

Even though different structured treatment schedules have been tested in clinical trials, a comparison with multiple trials is often difficult due to the heterogeneity of their experimental conditions. The clinical trials that tested the STI simulated in our work have different durations and different criteria for the enrollment of patients. Such diversity makes it difficult to compare their results with a single optimized schedule. In terms of initial conditions and treatment duration our simulations can be related to the clinical trial by Dybul *et al.* [16]. As in Dybul's clinical trial, our simulations have a treatment period of 48 weeks, enroll patients with similar CD4^+^ cells counts (CD4^+^ cells >300 cells/mm^3^) and test the STI schedule with 8 weeks of treatment and 4 weeks of interruption. The main difference is that in our simulations we use patients that have been never exposed to HAART and thus with a higher viral load whereas the clinical trial enrolled patients receiving a 3-drug HAART regimen with HIV RNA levels <500 copies/mL plasma for >6 months and <50 copies/mL at screening.

**Figure 4:**
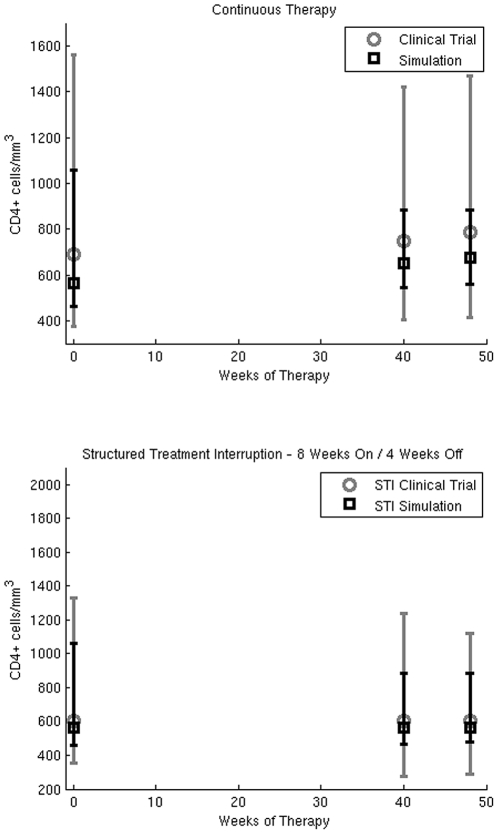
Model validation: CD4^+^ cell counts/mm^3^ at three different time points (Baseline, Week 40 and Week 48). In the upper panel we compare the median CD4^+^ cell counts of 250 virtual patients with the data of real patients from the continuous treatment arm of Dybul's clinical trial. In the lower panel we compare the median CD4^+^ cell counts of 250 virtual patients with the data of real patients from the STI arm of Dybul's clinical trial.

The parameters that control the pharmacodynamics of HIV in our model have been tuned by using clinical information from about twenty-two patients selected at the Clinical Department of the National Institute of Infectious Diseases “L. Spallanzani" in Rome [38]. No special tuning has been performed for the model parameters of the three STI schedules tested in the present work. For this reason we can validate our model results by comparing the simulations for both continuous treatment and the structured treatment interruption with data from Dybul's clinical trial. Those data consists of the median and range of CD4^+^ cell counts at baseline, at week 40 and at week 48 of the treatment period observed in 52 patients randomized in the continuous and STI arm of the clinical trial. In (Figure 4) we compare the simulations results with the values observed in the clinical trial. Simulated CD4^+^ cell counts are within the ranges observed in the clinical trial. In Table S1 we show the variation of immunological parameters for all the schedules tested in our virtual cohort. In the table we report the median value and the range observed in the virtual cohort at week 40 and week 48 of the treatment period.

**Figure 5:**
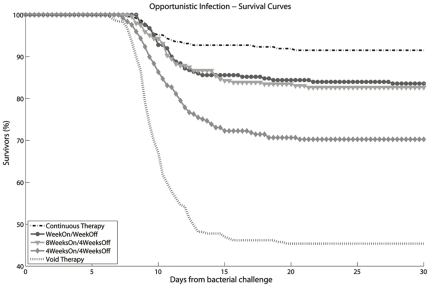
STI Survival curves for different STI strategies: percentage of survivors over time in a population of 250 HIV+ virtual patients. 30 days after bacterial challenge, survival rates are the following: Week On/Week Off STI 83.60%, 8 Weeks On/4 Weeks Off 82.66% and 4 Weeks On/4 Weeks Off 70.28%. Survival rates for continuous therapy and void therapy are 91.53% and 45.38% respectively.

Results of the STI strategies tested in clinical trials are shown in Figure 5. As observed in the clinical trials, all the STI strategies are associated with an increased number of deaths compared to the continuous treatment. The ratio of deaths associated to the “Week On/Week Off" strategy over that of continuous therapy is 1.95 at the end of the 30 days after opportunistic bacterial infection. The same ratio is 3.52 for the “4 Weeks On/4 Weeks Off" strategy and 2.05 for the “8 Weeks On/4 Weeks Off". We observe a ratio of deaths of 1.19 for the Optimal therapy and 2.14 for the Random therapy.

**Figure 6:**
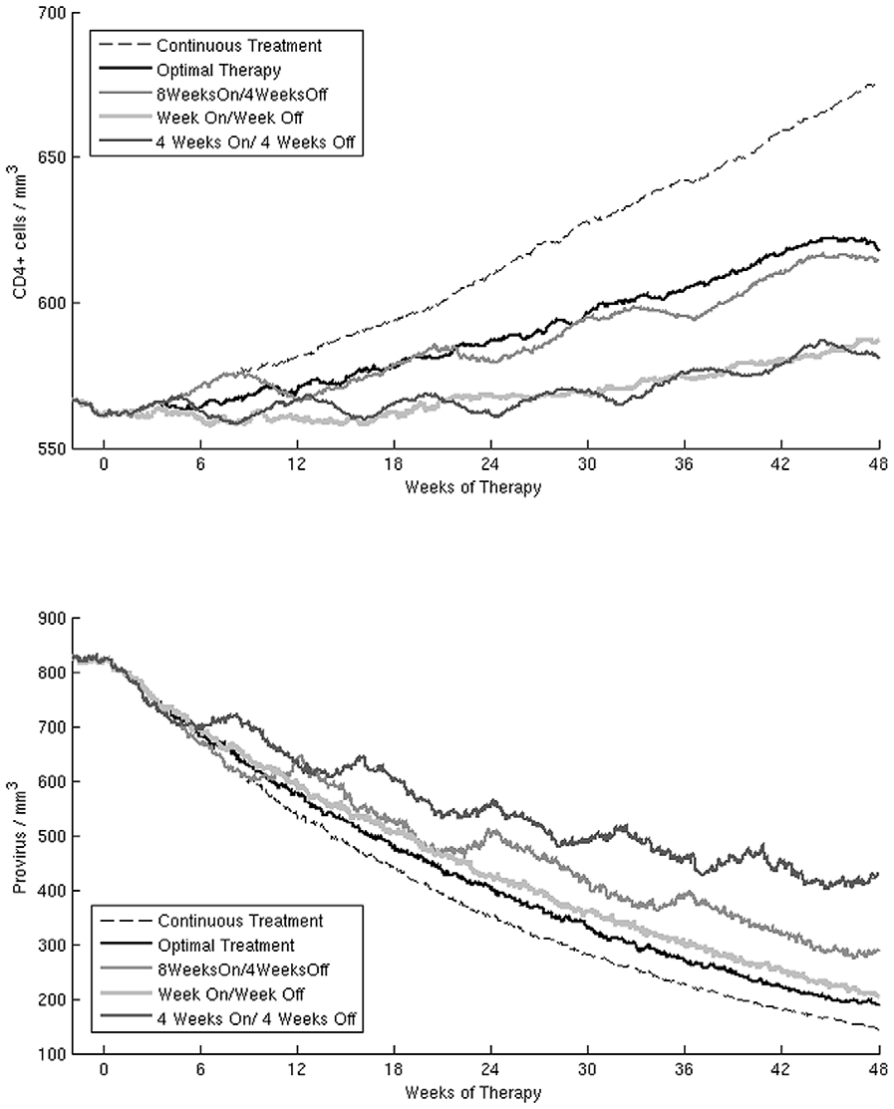
Simulation results. Median CD4^+^ cells count (upper panel) and provirus levels (lower panel) of the 250 virtual patients during the 48 weeks treatment period. In each panel we show the different STI tested in this study, the optimal therapy and the continuous therapy. The levels of provirus seem to be responsible for the failure of STI treatment as much as the CD4^+^ cells count. The “4 Weeks On/4 Weeks Off" schedule has the worst survival curve because of the high level of provirus even though it has a CD4^+^ cells count comparable to that of the “Week On/Week Off" STI.

The effects of the different STI on virological and immunological parameters are shown in (Figure 6). The parameters that show the strongest correlation with the survival of the virtual patients in the long term are CD4^+^ cell count and provirus levels. The levels of provirus seem to be responsible for the failure of STI treatment as much as the CD4^+^ cells count. The “4 Weeks On/4 Weeks Off " schedule has the worst survival because of the high level of provirus even though it shows a CD4^+^ cells count comparable to that of the “Week On/Week Off " STI. The difference in the ratio of deaths associated to “ 8 Weeks On/4 Weeks Off " and the optimal STI (2.05 versus 1.19) should be due to their difference in the level of provirus, since both schedules show comparable CD4^+^ cell counts. These results lead us to point out the importance of HIV reservoirs as one of the main causes of STI failure. As expected, a treatment interruption longer than 10 days leads to a viral rebound and a decrease in CD4^+^ cell count. We observe that the viral rebound is proportional to the duration of the interruption. Treatment interruptions of one or two weeks have a smaller viral rebound whereas for longer interruptions the viral load reaches levels comparable to the ones before treatment (data not shown).

**Figure 7:**
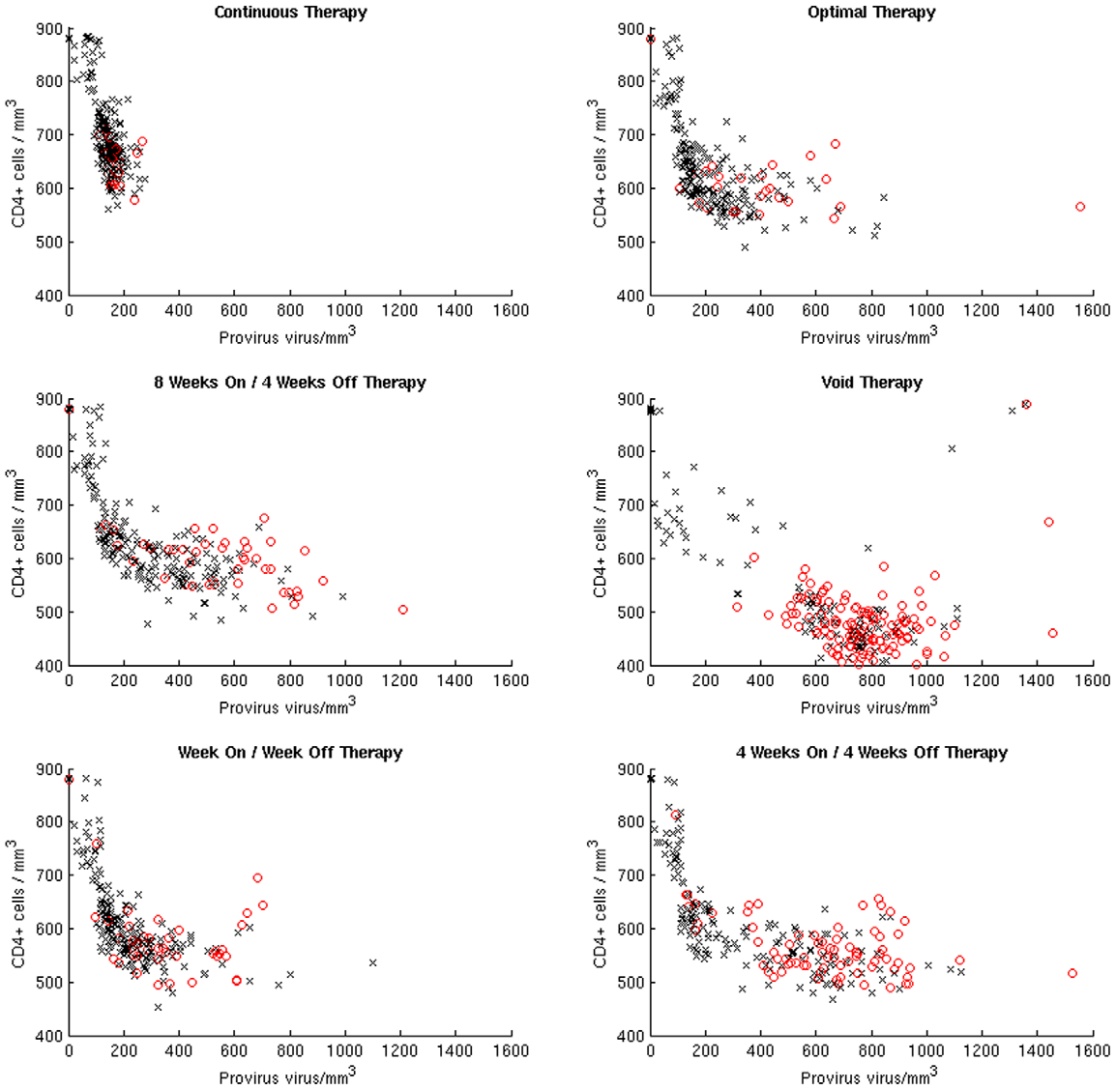
Survivors and Casualties. In each panel we show the CD4^+^ cell counts versus provirus of the 250 virtual patients at the end of the treatment period. The black markers indicate the patients that survived the opportunistic bacterial infection, whereas the red markers indicate the patients that died within 30 days of the bacterial infection.

For both optimal and continuous treatment the outcome of the opportunistic infection is correlated more to the level of provirus than to the level of CD4^+^ lymphocytes. For all other schedules it seems that both CD4^+^ and provirus are relevant in determining fatal outcomes of the bacterial infection. In Figure 7 we show the CD4^+^ cell counts versus provirus level of the 250 virtual patients for each of the treatment schedules tested in the present work. For each STI schedule we separate the 250 virtual patients in two groups (survivors and casualties) depending on the outcome of the opportunistic infection. We perform a Kolmogorov-Smirnov two-sample test to compare the distributions of the CD4^+^ cell counts at the end of the treatment period in both groups. The null hypothesis is that the CD4^+^ cell counts in the group of survivors and the one in the group of casualties are from the same distribution. For all the treatment schedules tested, except the optimal and continuous treatment, the null hypothesis is rejected with p-values between 1.1*10^−5^ and 1.6*10^−2^. Surprisingly for the optimal and continuous treatment the CD4^+^ cell count are not related to the outcome of the opportunistic infection (p-value 0.29 and 5.1*10^−2^). We perform the same test to compare the distributions of provirus in survivors and casualties. In this case the null hypothesis is rejected for all the schedules with p-values between 9.1*10^−12^ and 1.5*10^−2^.

## Discussion

The three STI strategies (“Week On/Week Off", “4 Weeks On/4Weeks Off" and “8 Weeks On/4Weeks Off") have lower performances compared to continuous treatment regardless of the emergence of drug resistance since the ratio of death associated to a clinically tested STI strategy over that of continuous treatment is between 1.95 and 3.52. Since in the model we don't allow HIV to develop resistance to cART drugs, the failure of STI seems to be inherent to the HIV infection dynamics, rather than caused by the emergence of drug resistant strains. We conclude that the amount of HIV reservoirs both in macrophages and latently infected resting/memory CD4^+^ T lymphocytes are sufficient to cause a failure of the STI schedules tested so far in clinical trials regardless of drug resistance. This observation may have relevant implications for the design of future treatment strategies. Indeed, efforts directed towards the reduction of the size of HIV reservoirs in humans, might facilitate the adoption of subsequent strategies aimed at reducing the antiretroviral treatment exposure, such as STI or treatments with less drugs [39].

We also show that, without the emergence of drug resistance, it is possible to find an optimized STI whose efficacy is close to that of a continuous treatment with a reduction of 40% in drug administration. In addition by showing the failure of the “8 Weeks On/4 Weeks Off" STI we show that adherence to the optimal treatment schedule is more important than the amount of drug taken over the treatment period. The efficacy of random therapies with the same amount of drug of the optimal treatment is sensibly lower than that of optimal treatment. Results indicate a ratio of death of 2.14 for the random treatment instead of 1.19 observed for the optimal. We conclude that the SA optimization was successful in identifying an optimal schedule of drug administration for the set of conditions studied in this work.

The result obtained in a previous work [32] on a shorter therapeutic period (6 months) and optimized by using a genetic algorithm indicated an optimized therapy with a week-on week-off pattern after a period of about 2 months of continuous therapy. The initial period of continuous therapy is not necessary according to our study, possibly because the immune system is not compromised enough to require an initial strong recovery phase. In the previous study the percentage of survivors for full and optimal therapy was comparable (34.1% and 30.5% respectively) whereas random and void therapy had lower performances (21.9% and 20.2%). The differences in the optimized treatments indicate that the best therapeutic strategy changes with the progressive damage that HIV infection inflicts on the immune system. Since at 3 years post infection the immune system is usually not severely compromised, in the present study the percentage of survivors is higher than the one observed in the previous study in which the therapy started almost 8 years after HIV infection. An interesting difference is the effect of random therapy. In our study we notice that a random therapy has less negative effects if the patient receives early (CD4^+^ cell counts >500 cell/mm^3^) cART. If treatment starts later random therapy has virtually no effect, as shown in [32]. This result indicates that unstructured interruptions have a less negative effect on the short term when the immune system is still healthy but further investigation is required to evaluate their effects in the long term.

The increased risk of opportunistic infections associated to provider-directed structured therapeutic interruptions led to abandoning this strategy in favor of the safety of a continuous treatment. Yet continuous treatment is not always possible as suggested by the proportion of patient-initiated unstructured interruptions. Since new studies suggesting the beneficial effects of early treatment may increase the duration of cART in a patient's life, a further growth of the already common unstructured interruptions can be foreseen. Moreover, so far, no clinical study has compared the risk of opportunistic infections associated with unstructured interruptions to the risk associated with STI. Finally, the sustainability of life-long antiretroviral therapy at global level, both from an economic and toxicity points of view, still represents a major challenge. For these reasons, exploring the mechanisms behind the failure of structured therapeutic interruptions and the possible optimal STI is still an open topic.

The ethical problems associated to further studies of STI in light of the previous failures make the use of modeling techniques appealing. The possibility of simulating STI *in silico* to predict the success or failure of a given STI strategy is a powerful tool that can support the design of clinical studies without having impact on human beings. The optimization of treatment interruptions is another clear example of the usefulness of modeling approaches to foster the understanding of complex problems.

For the HIV infection the lack of fully predictive animal models [40] makes it difficult to accurately validate the prediction of computational models. It is very difficult to address the ethical implications of testing computational predictions on humans, given the potential loss of human lives that could be caused by a wrong prediction. Yet, clinical trials are still needed and any tool that could be used to assist those trials should be considered.

Regardless of the difficulties in validating the predictions of our model, our results can be used to orientate qualitatively the design of clinical trials. *In silico* simulations could be used to predict if a STI schedule might fail because of the size of the HIV reservoir regardless of the emergence of drug resistance. In this way clinical trials could be directed toward schedules with a higher chance of success.

## Materials and Methods

### *In silico* approach

In this paper we adopt a two phases approach similar to the one used in Castiglione et al. 2007 [32] to study the existence of an optimal treatment that minimizes the drug administration without compromising the immune system response to HIV. Firstly we search for the optimal STI schedule (“optimal therapy") by using the simulated annealing optimization algorithm. In this phase, looking at the conditions of the immune system at the end of the therapeutic period, we evaluate the potentially administered therapeutic schedule by means of computer simulations. The SA algorithm is applied to search for the optimal schedule of cART interruptions that maximizes both the immune system recovery and viral control and, at the same time, minimizes the amount of drugs used in a therapeutic period. Secondly, once we have found the optimal schedule, we test it by challenging the immune system with an opportunistic infection simulated by introducing a bacterium in a group of 250 *virtual* patients. Therefore, we compare the survival curves of the different control groups with the survival curve of the optimal therapy. In the first control group patients have a continuous course of therapy (i.e., uninterrupted) for the whole period (“continuous therapy"). Patients of the second control group receive the same amount of medicine as in the optimal therapy, but the drugs are randomly administered in time (“random therapy"). The last control group consists of untreated patients (“void therapy"), that is, patients that do not receive therapy at all. Performing this test *in silico* allows us to use the same *virtual* patients for each group, whereas in clinical trials the control groups consist of different individuals. Such choice enables us to directly relate the survival of rates of each group to the effect of the different therapies.

In addition to the search for an optimal STI we also study the effects of STI strategies tested in clinical trials by comparing their effect on the HIV dynamics of the *virtual* patients over a period of 48 weeks of treatment. As control groups for this experiment we use groups of 250 virtual patients under continuous treatment and void treatment. Since the model does not include drug resistance, the outcomes of the STI strategies can be traced back to the dynamics of the immune response to the HIV virus and in particular to the action of antibody producing B lymphocytes and to the CD4^+^ and CD8^+^ T cells.

### Quantitative evaluation of STI

**Figure 8:**
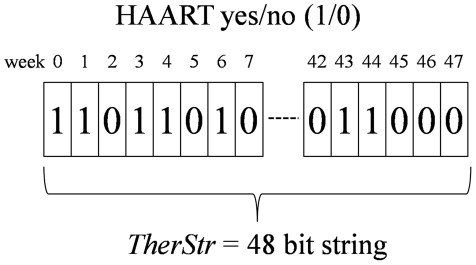
HAART schedule is represented as a 48-bit string. In the string, the j^th^ bit set to 1 means that during the j^th^ week the HAART is administered to the patient.

The simulation starts at time zero with an injection of HIV viral particles and the HIV infection progresses untreated for three years. At that point the disease is already in its chronic phase and we start a therapeutic period of 48 weeks. During the therapeutic period we administer the cART according to a given schedule represented by *TherStr*, a 48-bit-long string, where a 0 stands for a week without therapy (*i.e.,* an interruption) and a 1 stands for a full week of therapy (Figure 8). We assume the efficacy of the therapy to drop to zero during an interruption so that the HIV life cycle may progress unhindered through all its stages during the interruptions. Most cART regimens administer a daily cocktail of at least three drugs, commonly two RTIs and one PI. Although we could optimize the therapy on a daily basis, we decided to constrain the minimum administration/interruption period to one week. Note that this is not a fundamental limitation in our approach, but rather a practical one since no patient would be able to follow a one-year-long therapeutic schedule changing on a daily basis. In addition this choice allows a direct comparison with a previous study based on a genetic algorithm. Another constraint to the simulated cART dictated by medical practice is that RTI and PI drugs are always administered at the same time. At the end of the 48 weeks we measure the efficacy of the therapy by assigning a score given by our fitness function. At the beginning of the therapeutic period, the average CD4^+^ count measured in the virtual patients is about 500 cells per microliter.

To quantitatively evaluate the effect of a STI schedule of cART we define a fitness function F_i_ for the i^th^ virtual patient. The fitness function measures the health of the virtual patient's immune system as the outcome of a simulation and the amount of drugs received over the 48 weeks period. Since the whole immune/HIV dynamics depends on the therapy administered, the fitness score is a function of *TherStr*. F_i_ is the sum of three terms H_i_, D_i_ and Z_i_.

- H_i_ takes into account the fitness of HIV which is computed as the average of the sum of both virus contained in infected cells and free viral particles divided by the viral set point here defined as the sum of free virus and virus in infected cells at time the treatment starts t_s_;
- D_i_ is a measure of the fitness of the immune system and is given by the average of the ratio between the CD4^+^ count of the healthy individual (*i.e.,* CD4^+^ count at time = 0, just before the infection) and the CD4^+^ count during the treatment period;
- Z_i_ measures the amount of drugs used as the ratio between the number of weeks of active therapy and the 48 weeks of treatment.

In order to partially account for inter-patient variability, the optimization algorithm tries to minimize F, the average fitness score of eight virtual patients.
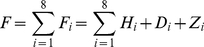
(1)where
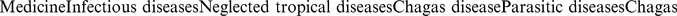
(2)

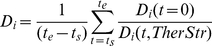
(3)

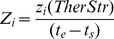
(4)In the formulas above t_s_ indicates the time the therapy starts (three years post infection); t_e_ the time the therapy ends (*i.e.,* 48 weeks after t_s_); H_i_(t,*TherStr*) is the sum of free virions and proviral HIV in infected cells; D_i_(t, *TherStr*) is the T helper cell count in the simulated space and z_i_(t, *TherStr*) is a function such that z_i_(t) = 1 if the therapy is active at time t, zero otherwise. Minimizing the fitness function implies decreasing the viral load and the amount of drugs used, whereas increasing the CD4^+^ cell count over a period of 48 weeks. C-ImmSim uses a time-step corresponding to 8 hours of real life.

### Simulated annealing optimization

To find the optimal schedule of the cART during the 48 weeks therapeutic period we use a Simulated Annealing algorithm [41]. To optimize the STI we perform *in silico* experiments on a test group of 8 virtual patients. The size of the test group is purposefully chosen equal to the number of cores available in each node of the computer cluster used to perform the optimization. This choice allows us to simulate in parallel the HIV infection on 8 virtual patients, by performing the computation on a single node.

We adapted the classic simulated annealing algorithm to take as input a therapeutic schedule in the form of a 48 bits string where each bit represents a week of therapy. We define an algorithm to update the therapeutic schedule that modifies the previous schedule by adding, removing or shifting 1 week of therapy (1 bit). By using this algorithm we assume that two consecutive configurations in the Markov chain have comparable fitness scores (*i.e.,* the energy of two consecutive configurations is comparable), which is a requirement for a correct annealing optimization.

The simulated annealing is characterized by a set of parameters described in Table S2. For the annealing we resort to a geometric cooling schedule [42]. We tested also logarithmic and adaptive cooling schedules. The logarithmic cooling was soon discarded due to the long computational times required and the adaptive did not result in a better convergence.

A similar optimization problem has been studied in the past by following an approach based on a genetic algorithm (GA) [32]. The GA study looked for the optimal therapy over a period of six months starting after 7.5 years post infection. One of the main differences between the present work and the previous one [32] is that we reduce the time between seroconversion and treatment from 7.5 to 3 years. This reduction allows considering the positive effect of early cART, but increases the difficulty of the optimization because the effect of cART on CD4 recovery is dampened by the healthier immune system at the time of treatment initiation. Another important difference is that we attempt to optimize the schedule over a longer period (close to 1 year) instead of just 24 weeks. Extending the therapeutic period to one year increases the complexity of the optimization problem. In the previous study the state space had 2^24^ possible configurations. In the present work we face a state space of 2^48^ configurations. Doubling the therapeutic period allows to test if the optimal STI can yield results similar to those observed in the shorter STI previously studied [32]. Clinical studies on STI indicated a reduction of the benefits over longer time periods [15], [16], [43]. By using an extended period of STI we have the chance to verify if we observe a similar behavior in the model.

**Figure 9:**
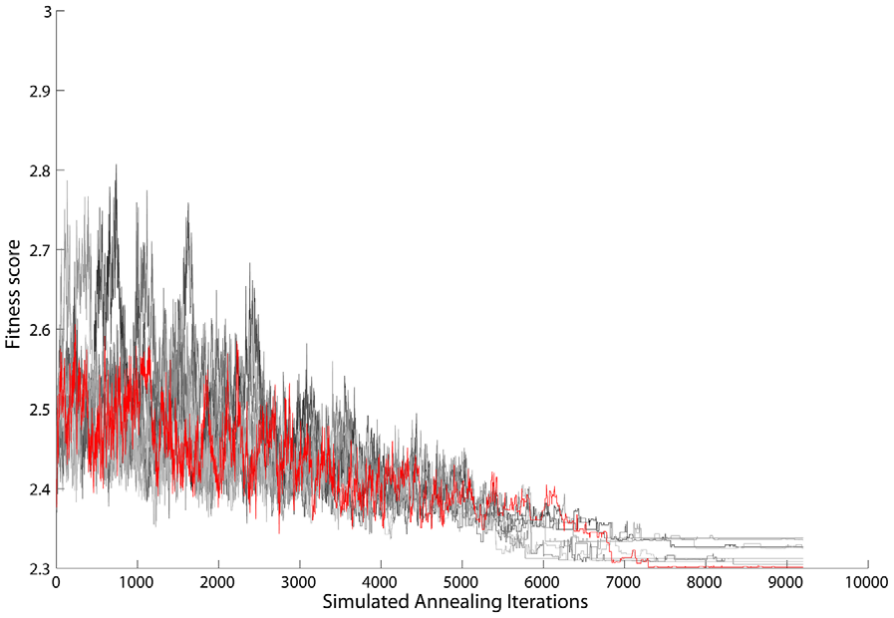
Simulated annealing algorithm: Fitness score progression during simulated annealing. Here we show 8 different annealing instances. The one corresponding to the lowest fitness is highlighted in red.

In each iteration of the optimization algorithm we evaluated the fitness function as the average among N = 8 virtual patients. The evaluation of the fitness of a given STI schedule required the simulation of 4 years of HIV infection dynamics in each individual patient. Most of the computational time is due to the C-ImmSim simulations, whereas the annealing algorithm has a relatively small impact on the computation time. The longest annealing optimizations use MAXITER equal to 27000 and take about 15 days to complete (Figure 9) using a L5520 eight-core processor having a clock rate equal to 2.26 GHz.

We explore stochastically the configuration space of 2^48^ different therapeutic schedules. We stop the optimization when the standard deviation of the fitness score for the last homogeneous Markov chains (*i.e.,* parameter MARKOVCHAIN in the Table S2 in Supporting Information) is lower than an arbitrary threshold set equal to 10^−4^. Each instance of the optimization algorithm results in an optimized schedule and a corresponding fitness score. We find several local optimal therapies with comparable fitness scores. As usual with most stochastic algorithms we do not have any warranty that a better solution could not be found by continuing the search.

## Supporting Information

File S1 **Description of the computational model used to simulate HIV infection in virtual patients.**
(DOC)

Table S1 **Variations of immunological and virological parameters: In the upper part of the table we report the data from Dybul's clinical trial** [**16**] **.** In the lower part of the table we show the results of our simulations. For each treatment schedule tested in our simulations we report the median value for the most important immunological and virological parameters. In parentheses we report the minimum and maximum values observed in the population of 250 virtual patients. For the viral load measurement ND indicates level of infectious virions below the detection level (<50 virions/ml).
(DOC)

Table S2 **Parameters of the simulated annealing algorithm.**
(DOC)
