# Supplementary material for: HIV Reservoirs and Immune Surveillance Evasion Cause the Failure of Structured Treatment Interruptions: A Computational Study
Source: PLoS One. 2012 Apr 27;7(4):e36108. doi: 10.1371/journal.pone.0036108 (PMC3338637; doi:10.1371/journal.pone.0036108)
Supplement: File S1 — Description of the computational model used to simulate HIV infection in virtual patients. (DOC) [file pone.0036108.s001.doc]

**S1.**

**Computational model**

The computational model we use, C-ImmSim, is a discrete-event stochastic agent-based model customized to simulate the innate (in part) and adaptive immune response to the HIV virus. Cells and molecules move on a 3D grid representing a lymph node. Upon collision the microscopic interactions among the immune cells and among cells and molecules are represented in the model by simple probabilistic rules. Extensive description of the model can be found in the literature [36]. For this work we used a volume of 4 microliters of lymphoid tissue corresponding to a fraction of a lymph node of average size. The number of simulated cells is comparable to that found experimentally in a corresponding volume of lymphatic tissue. The model computes the dynamics of both cellular and humoral immune response by simulating the complex interactions occurring among the main lymphoid cells (B, T cytotoxic, T helper and plasma cells), some myeloid cells (macrophages and dendritic cells), some cytokines and, obviously, the HIV virus. The phenomena of recognition and immune response against HIV are emergent properties of the microscopic interactions. A full account of the specific mechanisms of the HIV infection in untreated patients such as impairment of CD4^+^ T cell production, virus replication and virus mutation in target cells, have been previously published [35]. The model is able to reproduce the main characteristics of HIV infection with and without therapy as, for example, the viral rebound, the log-normal distribution of time to AIDS, the viral set point, and other stylized facts of the disease onset [34,35].

To simulate the effects of cART on the dynamics of the HIV disease we consider two classes of inhibitors: Reverse Transcriptase Inhibitors (RTIs) inhibiting the HIV reverse transcriptase enzyme and Protease Inhibitors (PIs) inhibiting the HIV protease enzyme. Inhibition of the DNA polymerase prevents the virus from carrying out the reverse-transcription of its RNA genome into DNA, effectively blocking the infection of new healthy cells. Inhibition of the protease enzyme blocks the maturation process of newly assembled virions, preventing them from being infectious. In the simulation, when anti-retroviral drugs are administered to the virtual patients, the HIV life cycle is modified as follows: when an infectious viral particle enters the cell cytoplasm, RTIs prevent the transition of the cell state from healthy to infected, since the virus is not able to reverse transcribe and integrate its genetic material into the host genome. On the other hand, the effect of PIs prevents the newly assembled viral particles from becoming infectious so that virions released by cells previously infected are not able to infect new cells. The dynamics of cART treatment implemented in this model is described in detail in a recent article that focuses on early *versus* late treatment strategies [42]. Drug resistance is not implemented in the version of the simulator used for the present study. This choice allows us to decouple the HIV mechanism behind the emergence of drug resistant strains from the mechanisms of hiding in cellular reservoirs and of epitope mutation to escape immune surveillance.
