# Supplementary material for: HIV Reservoirs and Immune Surveillance Evasion Cause the Failure of Structured Treatment Interruptions: A Computational Study
Source: PLoS One. 2012 Apr 27;7(4):e36108. doi: 10.1371/journal.pone.0036108 (PMC3338637; doi:10.1371/journal.pone.0036108)
Supplement: Table S1 — Variations of immunological and virological parameters: In the upper part of the table we report the data from Dybul's clinical trial [16] . In the lower part of the table we show the results of our simulations. For each treatment schedule tested in our simulations we report the median value for the most important immunological and virological parameters. In parentheses we report the minimum and maximum values observed in the population of 250 virtual patients. For the viral load measurement ND indicates level of infectious virions below the detection level (<50 virions/ml). (DOC) [file pone.0036108.s002.doc]

S2. Table 1. Variations of immunological and virological parameters

| **IN SILICO SIMULATION** | | | |
| --- | --- | --- | --- |
| **CD4+ T cell count cells/mm3** | WEEK1 median (range) | Week40 median (range) | Week 48: median (range) |
| Continuous Treatment | 562 (460-1060) | 653 (547-884) | 675 (559-883) |
| 8 Weeks On/4 Weeks Off Treatment | 562 (460-1060) | 607 (468-883) | 615 ( 477-884) |
| Optimal Treatment | 562 (460-1060) | 613 (487-884) | 620 (490-882) |
| Void Treatment | 561 (459-1069) | 492 (383-993) | 482 (378-950) |
| Random Treatment | 561 (459-1069) | 589 (488 – 883) | 599 (487-882) |
| 4 Weeks On/4 Weeks Off Treatment | 562 (460-1060) | 575 (473- 883) | 583 (470 -882) |
| Week On / Week Off Treatment | 562 (460-1060) | 579 (441-884) | 587 (450-882) |
| **VIRAL LOAD Log_10_ (virions/ml)** | WEEK1 median (range) | Week40 median (range) | Week 48: median (range) |
| Continuous Treatment | 3.59 (ND - 4.51) | ND (ND - 3.40) | ND (ND - 3.41) |
| 8 Weeks On/4 Weeks Off Treatment | 3.59 (ND - 4.51) | 1.85 (ND - 3.47) | 3.72 (ND - 4.99) |
| Optimal Treatment | 3.59 (ND - 4.51) | ND (ND - 4.50) | ND (ND - 5.16) |
| Void Treatment | 4.32 (ND - 5.18) | 4.28 (ND - 5.14) | 4.26 (ND - 5.06) |
| Random Treatment | 3.59 (ND - 4.19) | 3.18 (ND - 4.71) | 3.35 (ND - 4.64) |
| 4 Weeks On/4 Weeks Off Treatment | 3.59 (ND - 4.51) | 3.33 (ND - 4.38) | 4.01 (ND - 5.12) |
| Week On / Week Off Treatment | 3.59 (ND - 4.51) | 1.85 (ND - 4.38) | ND (ND - 4.36) |
| **Provirus virus/mm3** | WEEK1 median (range) | Week40 median (range) | Week 48: median (range) |
| Continuous Treatment | 817 (4 – 1699) | 193 (1 - 385) | 155 (1 – 352) |
| 8 Weeks On/4 Weeks Off Treatment | 817 (4 – 1699) | 341 (1 - 1140) | 282 (1 – 1151) |
| Optimal Treatment | 817 (4 – 1699) | 236 (1 - 1146) | 196 (1 – 1624) |
| Void Treatment | 817 (4 – 1699) | 529 (446 - 1516) | 725 (1 – 1494) |
| Random Treatment | 817 (4 – 1699) | 1313 (2 – 4155) | 274 (1 – 1224) |
| 4 Weeks On/4 Weeks Off Treatment | 817 (4 – 1699) | 474 (1 - 1482) | 409 (1 – 1528) |
| Week On / Week Off Treatment | 817 (4 – 1699) | 266 (1 – 1007) | 212 (1 – 883) |
