# Supplementary material for: HIV Reservoirs and Immune Surveillance Evasion Cause the Failure of Structured Treatment Interruptions: A Computational Study
Source: PLoS One. 2012 Apr 27;7(4):e36108. doi: 10.1371/journal.pone.0036108 (PMC3338637; doi:10.1371/journal.pone.0036108)
Supplement: Table S2 — Parameters of the simulated annealing algorithm. (DOC) [file pone.0036108.s003.doc]

S3.

Table2. Parameters of the simulated annealing algorithm

| Parameter | Value | Description | Notes |
| --- | --- | --- | --- |
| INIT_T | 0.2-0.045 | Initial Temperature | We tested several values of the initial temperature. In all cases INIT_T is high enough to accept most the moves in the state space at the beginning of the algorithm. The use of a high INIT_T ensures the system is not trapped in a capture basin. |
| MAXITER | 9000-27000 | Maximum number of iterations of the Metropolis algorithm | It is the maximum number of times C-ImmSim is launched. It is also one of the criteria that cause the termination of the SA. |
| MARKOVLENGTH | 5-10 | Length of the homogeneous Markov chain at a given temperature | The last configuration in the Markov chain should represent the configuration at thermal equilibrium for the given temperature. |
| MARKOVCHAINS | 10-20 | Number of Markov chains used to calculate the standard deviation of the fitness score |  |
| THRESHOLD | 10^-4^ | Value of the standard deviation below which the SA is stopped | It’s one of the stop criteria. The SA ends when the standard deviation of the fitness is lower than THRESHOLD. |
| NRUNS | 8 | It is the number of virtual patients over which the fitness score is averaged. | Corresponds to the number of cores used for the parallelization of the SA algorithm |
| C | 0.996-0.999 | Parameter characterizing the geometric cooling schedule for the SA | Geometric Cooling: T_k+1_ = C*T_k_ where T_k+1_ is the temperature of the next Markov chain and T_k_ is the temperature of the current one |
